# Supplementary material for: The impact of lag time to cancer diagnosis and treatment on clinical outcomes prior to the COVID-19 pandemic: A scoping review of systematic reviews and meta-analyses
Source: eLife. 2023 Jan 31;12:e81354. doi: 10.7554/eLife.81354 (PMC9928418; doi:10.7554/eLife.81354)
Supplement: Supplementary file 1. — The search was performed on 15 February 2021, limiting to publications from before the COVID-19 pandemic (1 January 2010–31 December 2019), with no restriction on publication language. [file elife-81354-supp1.docx]

Supplementary Table 1. Search strategy used to identify relevant systematic reviews and meta-analyses on the association between time to cancer diagnosis and treatment and outcomes of interest

| **Search term** | **Search number** | **Keywords/MeSH terms** |
| --- | --- | --- |
| Cancer | 1 | 'neoplasm'/exp OR (Neoplasms) OR (Cancer) OR (Cancers) OR (Neoplasia) OR (Neoplasm) OR (Tumors) OR (Tumor) OR (Malignancy) OR (Malignancies) OR (Malignant Neoplasms) OR (Malignant Neoplasm) OR (Neoplasm Benign) |
| Diagnosis & Treatment | 2 | (Diagnosis) OR (diagnose) OR (treat) OR (treatment) OR (therapy) OR (care) OR (screen) OR (surgery) OR (radiation therapy) OR (systemic therapy) OR (chemotherapy) OR (adjuvant chemotherapy) OR (adjuvant radiotherapy) OR (neoadjuvant chemotherapy) OR (neoadjuvant radiotherapy) |
| Exposure | 3 | (delay) OR (wait time) or (postpone) OR (interval) OR (deferred) OR (deferral) OR (time to surgery) OR (time to treatment) OR (waiting period) OR (patient delay) OR (provider delay) OR (doctor delay) OR (time to treatment initiation) OR (system delay) OR (doctor delay) OR (professional delay) OR (time interval) OR (postponement) OR (time to diagnosis) |
| Outcome | 4 | (outcomes) OR (recurrence) OR (survival) OR (mortality) OR (tumour progression) OR (postoperative complications) OR (oncologic outcomes) OR (disease free survival) OR (overall survival) OR (pathological complete response) OR (recurrence free survival) OR (local recurrence) OR (metastasis) OR (progression free survival) |
| Study type | 5 | (systematic review) OR (meta-analysis) OR (metanalyses) OR (systematic reviews) |
| Aggregation of search terms | 6 | 1 AND 2 AND 3 AND 4 AND 5 |
| Limits | 7 | Limit 6 to yr=“2010-2019” |

The search was performed on 15 February 2021, limiting to publications from before the COVID-19 pandemic (1 January 2010 - 31 December 2019), with no restriction on publication language.
