# Supplementary material for: The impact of lag time to cancer diagnosis and treatment on clinical outcomes prior to the COVID-19 pandemic: A scoping review of systematic reviews and meta-analyses
Source: eLife. 2023 Jan 31;12:e81354. doi: 10.7554/eLife.81354 (PMC9928418; doi:10.7554/eLife.81354)
Supplement: Supplementary file 2. [file elife-81354-supp2.docx]

Supplementary Table 2. Characteristics of included systematic reviews on the association between time to cancer diagnosis and treatment and clinical outcomes

| **First author (year)** | **Databases searched** | **Number of hits** | **Number of included studies** | **Total Number of participants** | **Countries in which studies were conducted** |
| --- | --- | --- | --- | --- | --- |
| Brasme (2012) (28) | EMBASE, Science Citation Index, Google Scholar | 6,412 | 98 | 22,619 | Not reported |
| van den Bergh (2013) (24) | PubMed and EMBASE | 4,950 | 17 | 34,517 | Not reported |
| Doubeni (2018) (30) | MEDLINE | 4,979 | 8 | 77,035 | Canada, France, USA |
| Foster (2013) (26) | MEDLINE, EMBASE | 2,111 | 16 | 2,628 | Not reported |
| Lethaby (2013) (29) | Medline, EMBASE, EMBASE Classic, Centre for Reviews and Dissemination databases, Cochrane Library, Medline In-Process, Other Non-Indexed Citations | 1,665 | 32 | 11,016 | France, Denmark, Israel, UK, Japan, Switzerland, Germany, Canada, USA, China, Brazil, Belgium, Italy, Hong Kong, Singapore, Turkey, South Africa, Nigeria, Sweden |
| Neal (2015) (20) | MEDLINE, MEDLINE in-process, EMBASE, Cumulative Index to Nursing and Allied Health Literature, PsychINFO, Cochrane Central Register of Controlled Trials, Database of Abstracts of Reviews of Effects, Cochrane Database of Systematic Reviews, Health Technology Assessment Database, NHS Economic Evaluation database. | 193,077 | 117 | 401,706 | UK, Italy, Spain, Netherlands, Denmark, Finland, France, Norway, Switzerland, Sweden, Germany, Poland, Austria, Belgium, Romania, Greece, Denmark, India, Japan, China, Hong Kong, Malaysia, South Korea, USA, Canada, Brazil, Turkey, Israel, Australia, New Zealand, Saudi Arabia, Libya, South Africa |
| Graboyes (2018) (23) | PubMed, EMBASE, Cumulative Index to Nursing and Allied Health Literature, Cochrane Library | Not reported | 5 | Not reported | Netherlands, USA, Canada, South Korea |
| Hansen (2018) (22) | PubMed, EMBASE, Cumulative Index to Nursing and Allied Health Literature, Cochrane Library | 3,259 | 5 | 13,514 | Denmark, USA, Canada, South Korea |
| Mattosinho (2019) (27) | PubMed/MEDLINE, Literatura Latino-Americana e do Caribe em Ciências da Saúde, Scientific Electronic Library Online | 434 | 9 | 1,560 | Brazil, Honduras, Chile, Argentina, Pero, México |
| Warren (2019) (25) | PubMed, EMBASE, MEDLINE | 575 | 10 | 30,298 | Not reported |
