# Supplementary material for: The impact of lag time to cancer diagnosis and treatment on clinical outcomes prior to the COVID-19 pandemic: A scoping review of systematic reviews and meta-analyses
Source: eLife. 2023 Jan 31;12:e81354. doi: 10.7554/eLife.81354 (PMC9928418; doi:10.7554/eLife.81354)
Supplement: Supplementary file 3. — AC, adjuvant chemotherapy; CI, confidence interval; DFS, disease-free survival; HR, hazard ratio; LR, local recurrence; OR, odds ratio; OS, overall survival; pCR, pathological complete response; RCT, randomized controlled trial; RR, risk ratio; SMM, smoldering multiple myeloma. Significant pooled risk estimates are bolded. -- indicates that subgroup and/or sensitivity analyses were not conducted or were not available. [file elife-81354-supp3.docx]

**Supplementary Table 3.** Characteristics of, and subgroup and/or sensitivity analysis reported by included meta-analyses on the association between time to cancer diagnosis and treatment and clinical outcomes

| **First author (year)** | **Databases searched** | **Number of hits** | **Number of included studies** | **Total number of participants** | **Subgroup and/or Sensitivity analyses** | | | **Countries in which studies were conducted** |
| --- | --- | --- | --- | --- | --- | --- | --- | --- |
|  |  |  |  |  | **Covariate** | **Response variable of interest** | **Pooled risk estimate [95% CI]**  **(model type, heterogeneity statistics I^2^ or Ri)** |  |
| Gomez (2009) (37) | MEDLINE, EMBASE, and ISI proceedings | Not reported | 9 | 1,895 | Cancer type | TNM (Oral) | **OR:1.47 [1.09–1.99]** (fixed-effects) | USA, Israel, Canada, Finland, Greece, Thailand, Japan, Brazil, UK |
|  |  |  |  |  |  |  | **OR:1.55 [0.96–2.51]** (random-effects, Ri = 0.55) |  |
|  |  |  |  |  |  | TNM (Oral delay >1 month) | **OR:1.69 [1.26–2.77]** (fixed-effects) |  |
|  |  |  |  |  |  |  | **OR:1.41 [0.73–2.75]** (random-effects, Ri = 0.80) |  |
|  |  |  |  |  | Study Quality | TNM (High-quality) | **OR: 1.40 [1.11–1.76] (fixed-effects)** |  |
|  |  |  |  |  |  |  | OR: 1.31 [0.83–2.07] (random-effects, Ri= 0.74) |  |
|  |  |  |  |  |  | TNM (low-quality) | OR: 1.01 [0.62–1.65] |  |
|  |  |  |  |  |  |  | OR: 1.14 [0.48–2.74] (random-effects, Ri= 0.39) |  |
|  |  |  |  |  | Follow-up period | TNM (≥ 10 year) | **OR: 1.40 [1.11–1.76]** |  |
|  |  |  |  |  |  |  | OR: 1.31 [0.83–2.07] (random-effects, Ri=0.74) |  |
|  |  |  |  |  |  | TNM (< 10 year) | OR: 1.01 [0.62–1.65] |  |
|  |  |  |  |  |  |  | OR: 1.14 [0.48–2.74] (random-effects, Ri= 0.67) |  |
|  |  |  |  |  | Stratification by site | TNM (Yes) | **OR: 1.51 [1.19–1.90]** |  |
|  |  |  |  |  |  |  | OR: 1.47 [0.93–2.32] (random-effects, Ri= 0.73) |  |
|  |  |  |  |  |  | TNM (No) | OR: 0.85 [0.55–1.31] |  |
|  |  |  |  |  |  |  | OR: 0.93 [0.49–1.79] (random-effects, Ri= 0.54) |  |
|  |  |  |  |  | Confounding adjustment (tobacco smoking, alcohol consumption) | TNM (Yes) | OR: 1.06 [0.83–1.35] |  |
|  |  |  |  |  |  |  | OR: 1.02 [0.72–1.43] (random-effects, Ri= 0.46) |  |
|  |  |  |  |  |  | TNM (No) | OR: 2.26 [1.54–3.32] |  |
|  |  |  |  |  |  |  | OR: 2.13 [0.97–4.67] (random-effects, Ri= 0.74) |  |
| Des Guetz (2010) (43) | PubMed | 42 | 11 | 17,645 | Excluding the 2 largest studies | Mortality | **RR:1.14 [1.04–1.25]** | Not reported |
|  |  |  |  |  | Cut-off delays between surgery and AC | Mortality | **RR:1.18 [1.13–1.23]** |  |
|  |  |  |  |  | Studies on Colon cancer | Mortality (Colon) | **HR: 1.25 [1.17-1.34]** |  |
|  |  |  |  |  |  | Mortality (Stage III colon) | **HR: 1.25 [1.18-1.33]** |  |
| Biagi (2011) (44) | MEDLINE, EMBASE, Cochrane Database of Systematic Reviews, Cochrane Central Register of Controlled Trials | 198 | 10 | 15,410 | Excluding the 2 largest studies | Mortality | **HR: 1.15 [1.10-1.22]** | Not reported |
|  |  |  |  |  | Excluding the 3 largest studies | Mortality | **HR: 1.13 [1.10-1.17]** |  |
|  |  |  |  |  | Excluding the 3 largest studies | Cancer-specific survival | **HR: 1.15 [1.10-1.19]** |  |
| Seoane (2012) (19) | MEDLINE, EMBASE, ISI Proceedings | 1,016 | 10 | 1,286 | Cancer type | Mortality (oral cancer) | RR: 1.00 [0.92-1.10] (fixed-effects) | Finland, Spain, USA, Denmark |
|  |  |  |  |  |  |  | RR: 1.27 [0.81–1.98] (random-effects, Ri = 0.94) |  |
|  |  |  |  |  |  | Mortality (pharynx cancer) | **RR: 1.68 [1.22-2.31]** (fixed-effects) |  |
|  |  |  |  |  |  |  | **RR: 1.69 [1.05–2.72]** (random-effects, Ri = 0.55) |  |
|  |  |  |  |  |  | Mortality (larynx cancer) | **RR: 1.05 [1.02-1.08]** (fixed-effects) |  |
|  |  |  |  |  |  |  | RR: 1.64 [0.91–2.96] (random-effects, Ri = 1.00) |  |
|  |  |  |  |  | Percentage of cases in stage III and IV | Mortality (≥ 60%) | **RR: 1.74 [1.30-2.33]** (fixed-effects) |  |
|  |  |  |  |  |  |  | **RR: 1.76 [1.21–2.54]** (random-effects, Ri = 0.37) |  |
|  |  |  |  |  |  | Mortality (<60%) | **RR: 1.04 [1.01-1.07]** (fixed-effects) |  |
|  |  |  |  |  |  |  | RR: 1.19 [0.99–1.44] (random-effects, Ri = 0.96) |  |
|  |  |  |  |  | Study design | Mortality (retrospective) | **RR: 1.09 [1.00-1.19]** (fixed-effects) |  |
|  |  |  |  |  |  |  | **RR: 1.57 [1.11–2.24]** (random-effects, Ri = 0.92) |  |
|  |  |  |  |  |  | Mortality (partially prospective) | **RR: 1.04 [1.01-1.07]** (fixed-effects) |  |
|  |  |  |  |  |  |  | RR: 1.34 [0.69–2.61] (random-effects, Ri = 1.00) |  |
|  |  |  |  |  | Primary care centres | Mortality | **RR: 1.50 [1.25-1.79]** (fixed-effects) |  |
|  |  |  |  |  |  |  | **RR: 1.77 [1.14–2.73]** (random-effects, Ri = 0.81) |  |
|  |  |  |  |  | Questionnaires | Mortality | **RR: 1.04 [1.01-1.07]** (fixed-effects) |  |
|  |  |  |  |  |  |  | RR: 1.04 [0.95–1.13] (random-effects, Ri = 0.84) |  |
|  |  |  |  |  | Study participants | Mortality (population-based) | RR: 1.02 [0.92-1.11] (fixed-effects) |  |
|  |  |  |  |  |  |  | RR: 1.16 [0.82–1.65] (random-effects, Ri = 0.89) |  |
|  |  |  |  |  |  | Mortality (hospital-based) | **RR: 1.05 [1.02-1.08]** (fixed-effects) |  |
|  |  |  |  |  |  |  | **RR: 1.67 [1.14–2.44]** (random-effects, Ri = 0.99) |  |
|  |  |  |  |  | Source of mortality data unknown | Mortality | **RR: 1.09 [1.00-1.19]** (fixed-effects) |  |
|  |  |  |  |  |  |  | **RR: 1.68 [1.13–2.49]** (random-effects, Ri = 0.94) |  |
|  |  |  |  |  | Source of data unknown | Mortality | **RR: 1.04 [1.01-1.07]** (fixed-effects) |  |
|  |  |  |  |  |  |  | RR: 1.17 [0.85–1.60] (random-effects, Ri = 0.99) |  |
|  |  |  |  |  | Adjusted for Sex | Mortality (yes) | **RR: 1.04 [1.01-1.07]** (fixed-effects) |  |
|  |  |  |  |  |  |  | RR: 1.16 [0.99–1.36] (random-effects, Ri = 0.94) |  |
|  |  |  |  |  |  | Mortality (no) | **RR: 2.77 [1.81-4.24]** (fixed-effects) |  |
|  |  |  |  |  |  |  | **RR: 2.77 [1.81–4.24]** (random-effects, Ri = 0.00) |  |
|  |  |  |  |  | Study quality | Mortality (study quality score ≥4) | **RR: 1.54 [1.28-1.86]** (fixed-effects) |  |
|  |  |  |  |  |  |  | **RR: 1.77 [1.14–2.75]** (random-effects, Ri = 0.81) |  |
|  |  |  |  |  |  | Mortality (study quality score <4) | **RR: 1.04 [1.012-1.07] (**fixed-effects) |  |
|  |  |  |  |  |  |  | RR: 1.04 [0.93–1.17] (random-effects, Ri = 0.89) |  |
| Yu (2013) (40) | PubMed, EMBASE, Cochrane Library, Web of Science | 1,157 | 7 | 34,097 | Stepwise exclusion | Mortality (1 study) | **HR: 1.17 [1.12-1.22]** | Italy, France, Denmark, USA |
|  |  |  |  |  |  | Mortality (2 studies) | **HR: 1.23 [1.12-1.34]** |  |
| Seoane (2015) (18) | MEDLINE, EMBASE, Web of Science | 653 | 10 | 1,023 | Study quality | TNM, T1, T2, T4, or T11, high quality | **RR: 2.44 [1.36–4.36]** (fixed-effects) | Canada, Thailand, Finland, Netherlands, UK, Argentina, Spain |
|  |  |  |  |  |  |  | **RR: 2.44 [1.36–4.36]** (random-effects, Ri = 0.00) |  |
|  |  |  |  |  |  | TNM, T1, T2, T4, or T11, low quality | **RR: 1.53 [1.26–1.86]** (fixed-effects) |  |
|  |  |  |  |  |  |  | **RR: 1.53 [1.12–2.08]** (random-effects, Ri = 0.55) |  |
| Wang (2015) (33) | PubMed, Cochrane Library, EMBASE | 3,053 | 15 | 4,431 | Time interval | pCR rate (5 weeks) | RR: 0.67 [0.40-1.12] | Not reported |
|  |  |  |  |  |  | pCR rate (6 weeks) | RR: 1.03 [0.76-1.42] |  |
|  |  |  |  |  |  | pCR rate (7 weeks) | **RR: 1.45 [1.18-1.78]** |  |
|  |  |  |  |  |  | pCR rate (8 weeks) | **RR: 1.49 [1.15-1.92]** |  |
|  |  |  |  |  |  | pCR rate (10 weeks) | RR: 0.83 [0.65-1.06] |  |
|  |  |  |  |  |  | pCR rate (12 weeks) | RR: 0.81 [0.60-1.08] |  |
| Gupta (2016) (31) | MEDLINE, EMBASE, Cochrane Register of Controlled Trials | Not reported | 11 included in primary analysis (34 included in review) | 79,616 (of the 34 studies) | Treatment sequencing vs non-sequencing studies | LR (sequencing) | RR: 1.08 [0.98-1.19] (fixed-effects) | Not reported |
|  |  |  |  |  |  | LR (non-sequencing) | **RR: 1.08 [1.01-1.15] (fixed-effects)** |  |
|  |  |  |  |  |  | Mortality (sequencing) | RR: 1.0 [0.94-1.06] (fixed-effects) |  |
|  |  |  |  |  |  | Mortality (non-sequencing) | RR: 0.98 [0.87-1.10] (fixed-effects) |  |
|  |  |  |  |  | Inclusion of all studies regardless of quality | LR | **RR: 1.07 [1.03-1.10]** |  |
|  |  |  |  |  |  | Mortality | **RR: 1.06 [1.04-1.07]** |  |
| Petrelli (2016) (34) | PubMed, EMBASE, Web of Science, Cochrane Library | 5,065 | 13 | 3,584 | -- | | | Not reported |
| Raphael (2016) (41) | MEDLINE, EMBASE | 1,326 | 14 | 56,269 | Study type | Mortality (RCT) | RR: 1.06 [0.97-1.16] (random effects) | Denmark, Italy, France, Canada, US, England, New Zealand |
|  |  |  |  |  |  | Mortality (institution-based cohort study) | **RR: 1.20 [1.00-1.44]** (random effects) |  |
|  |  |  |  |  |  | Mortality (population-based cohort study) | **RR: 1.43 [1.43-1.69]** (random effects) |  |
|  |  |  |  |  | Validity | Worsened DFS (high validity only) | **RR: 1.04 [1.00-1.08]** |  |
|  |  |  |  |  |  | Worsened DFS (not high validity) | RR: 1.06 [0.97-1.15] |  |
|  |  |  |  |  |  | Worsened DFS (high validity) | RR: 1.06 [0.99 -1.12] (random effects) |  |
|  |  |  |  |  |  | Worsened DFS (not high validity) | RR: 1.06 [0.97 -1.15] (random effects) |  |
| Du (2017) (36) | PubMed, EMBASE, and Cochrane Library | 1,914 | 13 | 19,652 | Stepwise exclusion | pCR | **RR: 1.25 [1.16-1.35]** | Not reported |
|  |  |  |  |  |  |  | **RR: 1.25 [1.16-1.35]** |  |
|  |  |  |  |  |  |  | **RR: 1.31 [1.07-1.60]** |  |
|  |  |  |  |  |  |  | **RR: 1.25 [1.16-1.35]** |  |
|  |  |  |  |  |  |  | **RR: 1.26 [1.16-1.36]** |  |
|  |  |  |  |  |  |  | **RR: 1.27 [1.17-1.37]** |  |
|  |  |  |  |  |  |  | **RR: 1.25 [1.16-1.35]** |  |
|  |  |  |  |  |  |  | **RR: 1.25 [1.16-1.36]** |  |
|  |  |  |  |  |  |  | **RR: 1.25 [1.16-1.35]** |  |
|  |  |  |  |  |  |  | **RR: 1.25 [1.15-1.35]** |  |
|  |  |  |  |  |  |  | **RR: 1.24 [1.15-1.35]** |  |
|  |  |  |  |  |  |  | **RR: 1.26 [1.17-1.36]** |  |
|  |  |  |  |  | Waiting interval | pCR (7-weeks) | **RR: 1.51 [1.20-1.89]** |  |
| Lin (2017) (38) | MEDLINE, EMBASE, Clinical Trials, Cochrane Central Register of Controlled Trials. | 2,622 | 5 | 1,016 | Stepwise exclusion | 2-year OS, 5-year OS, pCR, postoperative mortality, anastomotic leakage | -- | Italy, USA, Taiwan, France |
| Liu (2017) (46) | PubMed, Web of Science | 5,277 | 14 | 59,569 | Study design | Mortality (prospective)  (Categorical lag-time variable) | **HR: 1.22 [1.02-1.46] (random-effects, I^2^ = 48.7%)** | (Continent only provided) Asia, North America, Europe |
|  |  |  |  |  |  | Mortality (retrospective)  (Categorical lag-time variable) | **HR: 1.08 [1.01-1.15] (random-effects, I^2^ = 0%)** |  |
|  |  |  |  |  | Geographic location | Mortality (Asia)  (Categorical lag-time variable) | HR: 1.06 [0.80-1.38] (random-effects, I^2^ = NA) |  |
|  |  |  |  |  |  | Mortality (North America)  (Categorical lag-time variable) | HR: 1.11 [0.94-1.32] (random-effects, I^2^ = 87.7%) |  |
|  |  |  |  |  |  | Mortality (Europe)  (Categorical lag-time variable) | **HR: 1.25 [1.05-1.29] (random-effects, I^2^ = 21.6%)** |  |
|  |  |  |  |  | Number of cases | Mortality (<600)  (Categorical lag-time variable) | HR: 1.16 [0.83-1.62] (random-effects, I^2^ = 60.6%) |  |
|  |  |  |  |  |  | Mortality (≥600)  (Categorical lag-time variable) | **HR: 1.15 [1.02-1.29] (random-effects, I^2^ = 77.6%)** |  |
|  |  |  |  |  | Residual disease | Mortality (Yes)  (Categorical lag-time variable) | HR: 0.69 [0.30-1.60] (random-effects, I^2^ = NA) |  |
|  |  |  |  |  |  | Mortality (No)  (Categorical lag-time variable) | HR: 1.35 [0.51-3.57] (random-effects, I^2^ = NA) |  |
|  |  |  |  |  | Chemotherapy | Mortality  (Platinum-based)  (Categorical lag-time variable) | HR: 1.26 [0.98-1.63] (random-effects, I^2^ = 7.8%) |  |
|  |  |  |  |  |  | Mortality  (Platinum-based plus Taxane)  (Categorical lag-time variable) | HR: 1.17 [1.04-1.32] (random-effects, I^2^ = 65.9%) |  |
|  |  |  |  |  | FIGO stage | Mortality  (All)  (Categorical lag-time variable) | HR: 1.09 [0.93-1.28] (random-effects, I^2^ = 0%) |  |
|  |  |  |  |  |  | Mortality  (III–IV)  (Categorical lag-time variable) | **HR: 1.23 [1.07-1.42] (random-effects, I^2^ = 81.5%)** |  |
|  |  |  |  |  |  | Mortality  (I–II)  (Categorical lag-time variable) | HR: 0.78 [0.51-1.19] (random-effects, I^2^ = NA) |  |
|  |  |  |  |  | Adjustment for FIGO staging as potential confounder | Mortality  (Yes)  (Categorical lag-time variable) | **HR: 1.16 [1.04-1.30] (random-effects, I^2^ = 70.9%)** |  |
|  |  |  |  |  |  | Mortality  (No)  (Categorical lag-time variable) | HR: 0.92 [0.48-1.77] (random-effects, I^2^ = NA) |  |
|  |  |  |  |  | Adjustment for histology as a potential confounder | Mortality  (Yes)  (Categorical lag-time variable) | HR: 1.07 [0.91-1.26] (random-effects, I^2^ = 36.7%) |  |
|  |  |  |  |  |  | Mortality  (No)  (Categorical lag-time variable) | **HR: 1.26 [1.19-1.34] (random-effects, I^2^ = 0%)** |  |
|  |  |  |  |  | Adjustment for residual disease as a potential confounder | Mortality  (Yes)  (Categorical lag-time variable) | **HR: 1.22 [1.10-1.36] (random-effects, I^2^ = 23.5%)** |  |
|  |  |  |  |  |  | Mortality  (No)  (Categorical lag-time variable) | HR: 1.09 [0.89-1.34] (random-effects, I^2^ = 62.6%) |  |
|  |  |  |  |  | Study design | Mortality (prospective)  (Continuous lag-time variable) | **HR: 1.06 [1.00-1.12] (random-effects, I^2^ = 0%)** |  |
|  |  |  |  |  |  | Mortality (retrospective)  (Continuous lag-time variable) | HR: 1.04 [0.97-1.13] (random-effects, I^2^ = 69.1%) |  |
|  |  |  |  |  | Geographic location | Mortality (Asia)  (Continuous lag-time variable) | **HR: 1.15 [1.04-1.27] (random-effects, I^2^ = NA)** |  |
|  |  |  |  |  |  | Mortality (North America)  (Continuous lag-time variable) | **HR: 1.01 [1.00-1.02] (random-effects, I^2^ = 0%)** |  |
|  |  |  |  |  |  | Mortality (Europe)  (Continuous lag-time variable) | **HR: 1.06 [1.00-1.12] (random-effects, I^2^ = 0%)** |  |
|  |  |  |  |  | Number of cases | Mortality (<600)  (Continuous lag-time variable) | **HR: 1.07 [1.00-1.15] (random-effects, I^2^ = 44.2%)** |  |
|  |  |  |  |  |  | Mortality (≥600)  (Continuous lag-time variable) | **HR: 1.01 [1.0-1.02] (random-effects, I^2^ = 55.8%)** |  |
|  |  |  |  |  | Residual disease | Mortality (Yes)  (Continuous lag-time variable) | **HR: 1.09 [1.01-1.78] (random-effects, I^2^ = 0%)** |  |
|  |  |  |  |  |  | Mortality (No)  (Continuous lag-time variable) | HR: 0.98 [0.94-1.03] (random-effects, I^2^ = 0%) |  |
|  |  |  |  |  | Chemotherapy | Mortality  (Platinum-based)  (Continuous lag-time variable) | HR: 1.00 [0.90-1.11] (random-effects, I^2^ = NA) |  |
|  |  |  |  |  |  | Mortality  (Platinum-based plus Taxane)  (Continuous lag-time variable) | HR: 1.02 [0.99-1.05] (random-effects, I^2^ = 26.2%) |  |
|  |  |  |  |  |  | Mortality (NA)  (Continuous lag-time variable) | **HR: 1.15 [1.04-1.27] (random-effects, I^2^ = NA)** |  |
|  |  |  |  |  | FIGO stage | Mortality (All)  (Continuous lag-time variable) | HR: 1.03 [0.93-1.14] (random-effects, I^2^ = NA) |  |
|  |  |  |  |  |  | Mortality (III–IV)  (Continuous lag-time variable) | HR: 1.05 [0.99-1.11] (random-effects, I^2^ = 66.4%) |  |
|  |  |  |  |  | Adjustment for FIGO staging as potential confounder | Mortality  (Yes)  (Continuous lag-time variable) | HR: 1.05 [0.99-1.11] (random-effects, I^2^ = 66.4%) |  |
|  |  |  |  |  |  | Mortality  (No)  (Continuous lag-time variable) | HR: 1.03 [0.93-1.14] (random-effects, I^2^ = NA) |  |
|  |  |  |  |  | Adjustment for histology as a potential confounder | Mortality  (Yes)  (Continuous lag-time variable) | HR: 1.02 [0.99-1.05] (random-effects, I^2^ = 26.2%) |  |
|  |  |  |  |  |  | Mortality  (No)  (Continuous lag-time variable) | HR: 1.07 [0.94-1.23] (random-effects, I^2^ = 72.0%) |  |
|  |  |  |  |  | Adjustment for residual disease as a potential confounder | Mortality  (Yes)  (Continuous lag-time variable) | **HR: 1.07 [1.00-1.15] (random-effects, I^2^ = NA)** |  |
|  |  |  |  |  |  | Mortality  (No)  (Continuous lag-time variable) | HR: 1.04 [0.98-1.09] (random-effects, I^2^ = 54.6%) |  |
| Loureiro (2017) (39) | PubMed/MEDLINE, EMBASE, Thomson Reuters, Web of Science Core Collection, ASTRO and ESTRO proceedings of annual meetings (2000-2013) | 1,369 | 12 | 5,212 | Excluding studies without WT to RT period restrictions | Mortality | HR: 1.0 [0.90-1.12] | Spain, Canada, Brazil, USA, Germany |
| Usón (2017) (45) | PubMed | 239 | 12 | 12,056 | Stepwise exclusion | Mortality, 3-year | **OR: 1.17 [1.05-1.29] (fixed-effects)** | Not reported |
| Zhan (2017) (42) | PubMed, EMBASE, Web of Science, Cochrane library, ASCO meeting abstracts | 2,390 | 12 | 78,462 | Excluding the largest 2 studies | Mortality | **HR: 1.10 [1.08-1.12] (random-effects, I^2^ = 43.5%)** | Italy, France, Denmark, USA, UK, China |
|  |  |  |  |  | Publication year | Mortality (1999) | **HR: 2.15 [1.2-3.85] (random-effects, I^2^ = %NA)** |  |
|  |  |  |  |  |  | Mortality (2005) | HR: 0.00 [0.02-1.05] (random-effects, I^2^ = 0%) |  |
|  |  |  |  |  |  | Mortality (2006) | **HR: 1.22 [1.11-1.34] (random-effects, I^2^ = 0%)** |  |
|  |  |  |  |  |  | Mortality (2013) | **HR: 1.08 [1.06-1.10] (random-effects, I^2^ = NA)** |  |
|  |  |  |  |  |  | Mortality (2014) | **HR: 1.10 [1.06-1.15] (random-effects, I^2^ = 0%)** |  |
|  |  |  |  |  |  | Mortality (2015) | **HR: 1.32 [1.23-1.41] (random-effects, I^2^ = 0%)** |  |
|  |  |  |  |  |  | Mortality (2016) | **HR: 1.13 [1.08-1.19] (random-effects, I^2^ = 0%)** |  |
|  |  |  |  |  | Excluding the largest study | Worsened DFS | **HR: 1.09 [1.03-1.14]** |  |
| Wu (2018) (32) | PubMed, EMBASE, MEDLINE, Cochrane Library | 897 | 5 | 1,244 | -- | | | Not reported |
| Petrelli (2019) (35) | MEDLINE, EMBASE, Cochrane Library | 8,752 | 34 | 141,853 | -- | | | Canada, US, Sweden, Netherlands, UK, Brazil, Korea, Denmark, Taiwan, Italy, Japan, China |
|  |  |  |  |  |  |  |  |  |
|  |  |  |  |  |  |  |  |  |
|  |  |  |  |  |  |  |  |  |
|  |  |  |  |  |  |  |  |  |
| Zhao (2019) (21) | PubMed, EMBASE, Medline, Cochrane, ClinicalTrials.gov | 258 | 8 | 885 | Treatment type | Disease progression (Melphalan-prednisone) | **RR: 0.22 [0.08-0.64] (random-effects, I^2^ = 59%)** | Not reported |
|  |  |  |  |  |  | Disease progression (bisphosphonate) | RR: 1.00 [0.83-1.21] (random-effects, I^2^ = 0%) |  |
|  |  |  |  |  |  | Disease progression (immunomodulatory drug | **RR: 0.43 [0.31-0.59] (random-effects, I^2^ = 0%)** |  |
|  |  |  |  |  |  | Disease progression (Monoclonal antibody) | RR: 0.87 [0.36-2.07] (random-effects, I^2^ = NA) |  |
|  |  |  |  |  | Treatment type | Mortality (Melphalan-prednisone) | RR: 1.05 [0.82-1.35] (fixed-effects, I^2^ = 37%) |  |
|  |  |  |  |  |  | Mortality (bisphosphonate) | RR: 0.76 [0.18-3.29] (fixed-effects, I^2^ = NA) |  |
|  |  |  |  |  |  | Mortality (immunomodulatory drug) | RR: 0.63 [0.38-1.04] (fixed-effects, I^2^ = 34%) |  |
|  |  |  |  |  |  | Mortality (Monoclonal antibody) | RR: 0.73 [0.17-3.08] (fixed-effects, I^2^ = 33%) |  |
|  |  |  |  |  | High-risk SMM | Disease progression | **RR: 0.51[0.37-0.70] (fixed-effects, I^2^ = 47%)** |  |
|  |  |  |  |  | High-risk SMM | Mortality | **RR: 0.53 [0.29-0.97] (fixed-effects, I^2^ = 0%)** |  |

AC, adjuvant chemotherapy; CI, confidence interval; DFS, disease-free survival; HR, hazard ratio; LR, local recurrence; OR, odds ratio; OS, overall survival; pCR, pathological complete response; RCT, randomized controlled trial; RR, risk ratio; SMM, smoldering multiple myeloma

Significant pooled risk estimates are bolded.

--, indicate that subgroup and/or sensitivity analyses were not conducted or were not available.
